# Correction: Behavioral-state modulation of inhibition is context-dependent and cell type specific in mouse visual cortex

**DOI:** 10.7554/eLife.31708

**Published:** 2017-09-06

**Authors:** Janelle MP Pakan, Scott C Lowe, Evelyn Dylda, Sander W Keemink, Stephen P Currie, Christopher A Coutts, Nathalie L Rochefort

Pakan JMP, Lowe SC, Dylda E, Keemick SW, Currie SP, Coutts CA, Rochefort NL. 2016. Behavioral-state modulation of inhibition is context-dependent and cell type specific in mouse visual cortex. *eLife* **5**:e14985. doi: 10.7554/eLife.14985.

Published 23, August 2016

During the production process, Figure 2 – figure supplement 2 was incorrectly given the legend for Figure 2 – figure supplement 3 and vice versa.

The article has been corrected accordingly.

